# P-832. MeMed BV in Management of Adults Presenting with COPD Exacerbation to the ED: A Single-Center, Real-World Study

**DOI:** 10.1093/ofid/ofaf695.1040

**Published:** 2026-01-11

**Authors:** Eli Audi, Tanya Gottlieb, Asala Abu-Ahmad, Mirit Hershman-Sarafov

**Affiliations:** Department of Internal Medicine A, Bnai Zion Medical Center, Affiliated to the Rappaport Faculty of Medicine, Technion-Israel Institute of Technology, Haifa 3104802, Israel, Haifa, Hefa, Israel; MeMed, Tirat Carmel, HaZafon, Israel; Infectious Diseases Unit, Bnai Zion Medical Center, Rappaport Faculty of Medicine, Technion-Israel Institute of Technology, Haifa 3104802, Israel, Haifa, Hefa, Israel; Bnai Zion medical center, Haifa, Hefa, Israel

## Abstract

**Background:**

Distinguishing between bacterial and viral etiologies in emergency department (ED) patients remains a clinical challenge, particularly for COPD exacerbations.

MeMed BV® (MMBV) is an FDA-cleared host-protein test that provides a score (0-100) indicating bacterial (or bacterial co-infection) versus viral (or other non-bacterial) infections, with sensitivity and specificity >90%. This study examined MMBV’s integration into routine ED workflows for COPD exacerbations.

**Methods:**

A single center, real-world study, where MMBV was incorporated into ED guidelines for managing adult patients with COPD exacerbation. Clinicians were trained to consider MMBV scores as: < 35 (viral/other non-bacterial), 35–65 (equivocal), and >65 (bacterial or co-infection). In this preliminary analysis (April-September 2024), inclusion criteria were COPD diagnosis, age ≥18 and ED presentation with respiratory complaints. Main exclusion criteria were trauma or non-COPD-related exacerbations. Data were prospectively collected on patient demographics, medical history, lab results, treatment course, hospitalization, and ED revisits (within 7-days).

MMBV concordance was defined as not prescribing antibiotics for viral results and prescribing antibiotics for bacterial results. Outcomes were analyzed separately for admitted and discharged patients.

**Results:**

Analysis included 128 patients (median age 71, IQR: 59–83), with 79.7% aged ≥65 and 32.0% female. MMBV results were bacterial in 60 (46.5%) patients, equivocal in 26 (20.1%), and viral in 43 (33.3%).

Twenty-two patients (17.1%) were discharged from the ED: 3 had bacterial results (all prescribed antibiotics), 6 had equivocal and 13 had viral results (none prescribed antibiotics); representing 100% MMBV concordance. None of the patients with non-equivocal MMBV results had unplanned ED revisits.

Among 106 admitted patients, 57 had bacterial results (70.2% received antibiotics), 20 had equivocal results (40.0% received antibiotics), and 29 had viral results (17.2% received antibiotics); representing 75.3% MMBV concordance.

**Conclusion:**

Incorporating MMBV into routine ED care for COPD exacerbations is associated with targeted antibiotic use and safe discharges.

**Disclosures:**

Tanya Gottlieb, PhD, MeMed Diagnostic: Employee

